# Overexpression of Neuroglobin Promotes Energy Metabolism and Autophagy Induction in Human Neuroblastoma SH-SY5Y Cells

**DOI:** 10.3390/cells10123394

**Published:** 2021-12-02

**Authors:** Valeria Manganelli, Illari Salvatori, Michele Costanzo, Antonella Capozzi, Daniela Caissutti, Marianna Caterino, Cristiana Valle, Alberto Ferri, Maurizio Sorice, Margherita Ruoppolo, Tina Garofalo, Roberta Misasi

**Affiliations:** 1 Department of Experimental Medicine, Sapienza University of Rome, 00161 Rome, Italy; valeria.manganelli@uniroma1.it (V.M.); antonella.capozzi@uniroma1.it (A.C.); daniela.caissutti@uniroma1.it (D.C.); maurizio.sorice@uniroma1.it (M.S.); roberta.misasi@uniroma1.it (R.M.); 2 Fondazione Santa Lucia IRCCS, c/o CERC, 00143 Rome, Italy; illari.salvatori@uniroma1.it (I.S.); c.valle@hsantalucia.it (C.V.); alberto.ferri@cnr.it (A.F.); 3 Department of Molecular Medicine and Medical Biotechnology, University of Naples Federico II, 80131 Naples, Italy; michele.costanzo@unina.it (M.C.); marianna.caterino@unina.it (M.C.); margherita.ruoppolo@unina.it (M.R.); 4 CEINGE–Biotecnologie Avanzate S.C.Ar.L, 80145 Naples, Italy

**Keywords:** neuroglobin, mitochondria, autophagy, energy metabolism, SH-SY5Y neuroblastoma cells, label-free proteomics

## Abstract

Neuroglobin (NGB) is an O_2_-binding globin mainly expressed in the central and peripheral nervous systems and cerebrospinal fluid. Previously, it was demonstrated that NGB overexpression protects cells from hypoxia-induced death. To investigate processes promoted by NGB overexpression, we used a cellular model of neuroblastoma stably overexpressing an NGB-FLAG construct. We used a proteomic approach to identify the specific profile following NGB overexpression. To evaluate the role of NGB overexpression in increasing energetic metabolism, we measured oxygen consumption rate (OCR) and the extracellular acidification rate through Seahorse XF technology. The effect on autophagy induction was evaluated by analyzing SQSTM1/p62 and LC3-II expression. Proteomic analysis revealed several differentially regulated proteins, involved in oxidative phosphorylation and integral mitochondrial proteins linked to energy metabolism. The analysis of mitochondrial metabolism demonstrated that NGB overexpression increases mitochondrial ATP production. Indeed, NGB overexpression enhances bioenergetic metabolism, increasing OCR and oxygen consumption. Analysis of autophagy induction revealed an increase of LC3-II together with a significant decrease of SQSTM1/p62, and NGB-LC3-II association during autophagosome formation. These results highlight the active participation of NGB in several cellular processes that can be upregulated in response to NGB overexpression, playing a role in the adaptive response to stress in neuroblastoma cells.

## 1. Introduction

Neuroglobin (NGB) is an O_2_-binding globin which is expressed in vertebrates mainly in the central and peripheral nervous systems and cerebrospinal fluid [1]. NGB reversibly binds oxygen with an affinity higher than that of hemoglobin, stores oxygen to supply cells and plays an important neuroprotective role [2,3]. Notably, NGB is physically and functionally related to mitochondrial functions [4]. Indeed, NGB is sensitive to changes in O_2_ availability and may play a role in ATP production [5]. Furthermore, this protein intervenes by inhibiting the intrinsic pathway of apoptosis, interfering with the release of cytochrome c (Cytc) from the mitochondria [6]. NGB overexpression in cortical, hippocampal neurons and human neuroblastoma cells protects from hypoxia-induced death, while NGB knockdown in cortical neurons exposes them to hypoxia and oxidative stress [7,8,9,10]. NGB is variously distributed in the cell, has been found in the cytosol, but also in the nucleus and mitochondria [11,12,13,14,15], a preferential localization site when NGB is overexpressed [13,16]. Regardless of the type of damage, the protective role of NGB appears to depend on two specific conditions: its overexpression and mitochondrial localization [16]. The redox state of the cell is strongly influenced by reactive oxygen species (ROS) which are by-products of aerobic metabolism [17]. In fact, while an excessive level of ROS involves an intracellular toxicity with processes of peroxidation, DNA damage and consequent cell death, low concentrations of ROS have a trophic action promoting the differentiation and cell growth. As a consequence, the balance between pro- and anti-oxidant compounds may greatly affect cell fate. Many papers reported the close relationship between NGB and oxidative stress in the nervous system; in fact, in conditions of high ROS, NGB demonstrates neuroprotective capacity both in vitro and in vivo [18,19,20,21,22]. To define the cytoprotective role of NGB, several mechanisms have been proposed [6,13,23,24,25]: NGB can act as a ROS/reactive nitrogen species (RNS) scavenger; with consequent antioxidant effect [23,24], it can play a role in the apoptotic pathway [6], or even regulate some pathways involved in cell survival [13]. In our previous study, NGB overexpression also showed a protective effect against 1-methyl-4-phenylpyridinium (MPP+) induced cytotoxicity in SK-N-B-E2 cells [16]. However, the possible interaction of NGB with various other molecules, either cytosolic or mitochondrial [26], broadens the horizons of the potential functional roles of NGB.

Autophagy is known to be one of the physiological mechanisms that the cell uses in survival strategies; therefore, it participates in the maintenance of homeostasis by carrying out a cytoprotective role [27,28] and NGB has already been challenged as an inducer of autophagy machinery [29]. Functional mitochondria are essential for the maintenance of cell integrity and reactivity [30,31,32], while dysfunctional mitochondria have severe cellular consequences on energy supply and these dysfunctions are clearly linked to diseases. In this context, alterations in mitochondrial metabolism have been widely described in metabolic disorders related to different pathologies [33], and a growing body of data showed a pivotal role of mitochondrial disfunction in Neurodegenerative Diseases (ND) [34]. For this reason, mitochondrial dysfunctions in ND may represent a potential therapeutic target [35,36]. Several pathogenic mechanisms take advantage by the overcoming of all protein quality control systems or, vice versa, by being the result of an altered function of systems, such as ubiquitin/proteasome and autophagy/lysosomes [37]. Mitochondrial homeostasis is therefore crucial for energy metabolism and for the well-being of the cell. The well-being of the mitochondria, in turn, depends on the correct folding of functional proteins and their turnover. In order to investigate processes promoted by NGB overexpression, here we used a cellular model of neuroblastoma that stably overexpresses an NGB-FLAG protein construct. Since human dopaminergic neurons are difficult to acquire and sustain as primary cells, established neuronal cell models are often used in research. Many dopaminergic cell lines lend themselves to this type of experimentation, including PC12 cells, a neuroendocrine cell line of rat pheochromocytoma, which has also been widely used in therapeutic [38] and oxidative stress trials [39,40] in neurodegeneration. However, one of the main advantages of working with SH-SY5Y cells is their human origin. This ensures not only a model of human gene and protein expression, but also the expression of the human form of proteins relevant for the disease [41,42].

**Figure 1:**
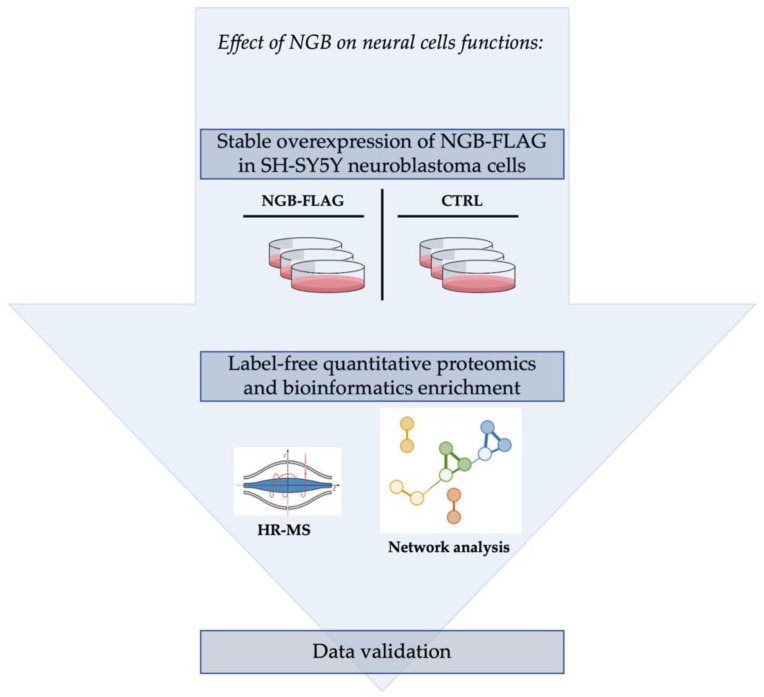
Schematic workflow of the experimental procedures performed in the current manuscript. Proteomic and bioinformatic analyses followed *NGB* overexpression in SH-SY5Y cells. Technical and functional experiments were performed to validate and support the data obtained from proteomic investigation. CTRL = control cells; HR-MS = High Resolution-Mass Spectrometry; NGB-FLAG = cells overexpressing NGB-FLAG protein.

In the current paper, a proteomic investigation was performed on NGB-FLAG cells using a shotgun label-free quantitative approach. An explicative workflow that schematically describes the experimental plan followed to discover the effects of overexpressed NGB on neural cells functions is reported in Figure 1. Globally, our proteomic and bioinformatic analyses revealed some altered processes in SH-SY5Y cells, such as transport, cytoskeleton organization and bioenergetic pathways, that may be linked to NGB function [43]. Furthermore, functional validation of our data revealed that neural cells possibly present a positive regulation of the energy metabolism, mitochondrial health, and lysosomal pathways. Therefore, the turnover of mitochondria could be correlated on the one hand with the mitochondrial mass and on the other with cell survival.

## 2. Materials and Methods

### 2.1. Cell Culture and Transfection

Human SH-SY5Y neuroblastoma cells (ATCC, LGC Standards S.r.l., Milan, Italy) were grown in DMEM/F12–Dulbecco’s Modified Eagle Medium: Nutrient Mixture F-12 (Thermo Fisher Scientific, Waltham, MA, USA) supplemented with 10% fetal calf serum (FCS), 10 mg/mL streptomycin and 100 units/mL penicillin in humified 5% CO_2_ atmosphere at 37 °C. The stable expression of vector was obtained with transfection through Lipofectamine Plus reagent (Invitrogen, Waltham, MA, USA) according to the manufacturer’s instruction, as previously described [44]. After 3 weeks of selection with 400 μg/mL G418 (Gibco, Waltham, MA, USA), single clones were isolated and screened according to their capability to express human NGB fused with 3XFLAG at C-Term (NGB-FLAG). The wild type SH-SY5Y neuroblastoma cells or SH-SY5Y cells transfected with an empty construct were used and control (CTRL).

### 2.2. Plasmid Construction

According to Garofalo et al. [16], we subcloned the human NGB ORF (NCBI Reference Sequence: NM_021257.3) using the forward primer 5′-AAAAAGATATCATGGAGCGCCCGGAGCCCGAG-3′ and the reverse primer 5′-AAAACTCGAGCTCGCCATCCCAGCCTCGACT-3′, by conventional PCR techniques from NGB-pCMV6-XL5 (Origene Technologies, Inc., Rockville, MD, USA). The resulting PCR fragment was inserted into *Eco*RV/*Xho*I restriction site of pcDNA3.1-FLAG (Invitrogen, USA). The plasmid construction was evaluated with automated sequencing.

### 2.3. Sample Preparation and LC-MS/MS Analysis

Cell samples were collected and processed as described for proteome extraction, quantization and digestion [45,46,47]. Briefly, six biological cell replicates per condition were lysed in RIPA buffer (Sigma-Aldrich, St. Louis, MO, USA) applying mechanical homogenization with TissueLyser II homogenizer (Qiagen, Duesseldorf, Germany). Lysates were treated with 1% Benzonase (E8263-5KU, Sigma-Aldrich, St. Louis, MO, USA) in 2 mM MgCl_2_ at 37 °C for 30 min, and then centrifuged at 18,000 rpm for 30 min at 4 °C to discard membranes, cell debris and nucleic acids. Digestion of proteomes was performed at 47 °C for four hours on S-Trap^TM^ micro spin columns (Protifi, Huntington, NY, USA), using trypsin (Promega, Madison, WI, USA) 1:25 per 50 µg of protein extracts. Eluted peptides were injected onto a liquid chromatography-tandem mass spectrometry (LC-MS/MS) system, constituted by an EASY-nLC^TM^ coupled with a LTQ-Orbitrap XL mass spectrometer (Thermo Scientific, Bremen, Germany) [48].

### 2.4. LFQ Proteomics Analysis and Bioinformatics Enrichment

Quantitative analysis of proteomics data was carried out by means of the LFQ (Label-free Quantification) approach using the MaxQuant software (version 1.6.17.0, MPI of Biochemistry, Martinsried, Germany), set as previously reported [43,49]. In addition, Perseus software, (version 1.6.14.0, MPI of Biochemistry, Martinsried, Germany) was employed for proteomic quality control and statistic comparative analysis [50,51]. The dataset matrix was reduced filtering out proteins for which valid values were found in less than the 50% of the samples. The imputation of missing values was done replacing by random numbers drawn from a normal distribution with width = 0.5 and down shift = 1.8. The comparison between the NGB-FLAG and CTRL conditions was carried out selecting proteins with values of S0 = 0.5 and *t*-test based on (False Discovery Rate) FDR = 0.01. The quantitative difference in the abundance of the statistically significant proteins was calculated as difference of the average log_2_ protein LFQ intensities of NGB-FLAG and CTRL samples. Thus, the differential dataset was graphically reported as volcano plot. Multivariate statistical analysis was carried out using MetaboAnalyst 5.0 software (https://www.metaboanalyst.ca accessed on 27 October 2021). In particular, the Principal Component Analysis (PCA) was used to assess the statistic segregation of the two compared groups [52,53]. Finally, the bioinformatic treatment of proteomics data was conducted using FunRich (version 3.1.4) (http://www.funrich.orgaccessed on 27 October 2021) to reveal the functional enrichment of biological processes, graphically depicted in the form of Doughnut chart [54]. Moreover, a cluster enrichment analysis was performed in STRING (version 11.0) (https://string-db.org accessed on 27 October 2021) to determine functional clusters in the protein–protein interaction (PPI) network produced using the differential dataset [55]. Western blot analysis for the validation of VDAC1 protein dysregulation was performed according to published procedures [49]. As primary antibody, an anti-VDAC pAb (Sigma-Aldrich, St. Louis, MO, USA) was used in 1% milk in PBS with 0.05% Tween-20 and incubated O/N at 4 °C. The primary antibody for the detection of GAPDH (Santa Cruz Biotechnology, Dallas, TX, USA) normalizing protein was incubated for 1 h at RT in the same milk solution. Three Western blot experiments were performed independently and data were averaged, with subsequent Student’s *t*-test for NGB-FLAG versus CTRL significant at *p*-value < 0.05.

### 2.5. Bioenergetic Analysis

By Seahorse XF96e Analyzer (Seahorse Bioscience—Agilent, Santa Clara, CA, USA), we analyzed the mitochondrial function and the energy phenotype of cells. The cells were plated at the density of 5 × 10^4^ and were incubated with growth medium for 24 h. Mitochondrial stress test and Real-Time ATP rate assay was carried out according to Agilent’s recommendations. For the Mitochondrial stress test, the growth medium was replaced with XF test medium (Eagle’s modified Dulbecco’s medium, w/o glucose, pH = 7.4; Agilent Seahorse) supplemented with 1 mM pyruvate, 10 mM glucose and 2 mM L-glutamine. Before the assay, the cells were incubated without CO_2_ at 37 °C for 1 h to allow the pre-equilibration with the assay medium. The test was performed by measuring at first the baseline oxygen consumption rate (OCR), followed by sequential OCR measurements obtained with injection of oligomycin (1.5 µM), carbonyl cyanide 4-(trifluoromethoxy) phenylhydrazone (FCCP) (1 µM) and Rotenone (0.5 µM) + Antimycin A (0.5 µM). This allowed to characterize the key parameters of the mitochondrial function including basal respiration, calculated as baseline OCR before oligomycin addition; after oligomycin addition, the ATP-linked respiration was obtained from the difference between the basal respiration and the minimal respiration. The maximal respiration was obtained as OCR after FCCP addition. On the other hand, in the real-Time ATP rate assay, the growth medium was replaced with XF test medium (Eagle’s modified Dulbecco’s medium, w/o glucose, pH = 7.4; Agilent Seahorse) supplemented as described above. The test was performed by measuring the baseline oxygen consumption rate (OCR), followed by sequential OCR measurements through the injection of oligomycin (1.5 µM) and (1 µM) and Rotenone (0.5 µM) + Antimycin A (0.5 µM). This allowed to characterize the bioenergetic parameters and the production of cellular ATP (ATP production rate) related to the conversion of glucose to lactate in the glycolytic pathway (glycoATP Production Rate) and to mitochondrial oxidative phosphorylation (mitoATP Production Rate). Accordingly, the ratio between mitoATP Production Rate and glycoATP Production Rate is considered as a valuable parameter to detect changes and differences in the metabolic phenotype OCR values. The assays were normalized to the total number of cells for each well and analyzed with XFe Wave software (Santa Clara, CA, USA).

### 2.6. DNA-RNA Isolation and Real Time qPCR

The content of mitochondria in the cells was performed with quantitative real-time PCR. Mitochondrial and genomic DNA were isolated according to Miller [56], using the PK, whereas total RNA was extracted with Trizol and retro-transcribed with a SensiFASTtm cDNA synthesis kit (Bioline, London, UK). Mitochondrial DNA content and mRNA expression levels were evaluated by Real Time qPCR reactions using Light Cycler 480 SYBR Green System (Roche ETC, Basel, Switzerland). Cp values of ND5, ND2 and NRF1 were calculated using the ‘second derivative max’ algorithm of the Lightcycler software (Roche Basel, Switzerland). Relative mitochondrial DNA content was normalized with the genomic genes TNF-alfa and Interleukin 2 (IL2), whereas relative mRNA expression values were normalized with the housekeeping gene TATA box binding protein. Primers sequences are in Table 1.

### 2.7. Analysis of LC3-II and SQSTM1/p62 Levels by Immunoblotting

SH-SY5Y cells, SH-SY5Y cells transfected with an empty construct (CTRL) and stably transfected SH-SY5Y-FLAG-NGB cells were lysed in cold lysis buffer, containing 1% Triton X-100 (Bio-Rad, Segrate, Milan, Italy), 10 mM Tris-HCl, pH = 7.5, 150 mM NaCl, 5 mM EDTA, 1 mM Na_3_VO_4_ (Sigma Aldrich) and 75 U of aprotinin (Sigma) for 20 min at 4 °C. The lysate was centrifuged for 5 min at 1300× *g* to discard nuclei and large cellular debris. Then, immunoblotting analysis was employed as reported above. The membranes were subsequently probed with rabbit anti-LC3 pAb (Novus Biologicals, Centennial, CO, USA), with rabbit anti-SQSTM1/p62 mAb (Cell Signaling Technology, Danver, MA, USA) or with anti-ACTB (actin, β) mAb (Sigma). Bound antibodies were visualized with horseradish peroxidase (HRP)-conjugated anti-rabbit IgG (Sigma) or anti-mouse IgG (Sigma) and immunoreactivity assessed by chemiluminescence reaction, using the ECL western detection system. Densitometric scanning analysis was performed by Mac OS X (Apple Computer International, Cupertino, CA, USA), using NIH Image, Version 1.62 software (National Institutes of Health; Bethesda, MD, USA).

### 2.8. Autophagy Induction

For autophagy induction, SH-SY5Y cells transfected with an empty construct or stably transfected SH-SY5Y-FLAG-NGB cells were starved by culturing under condition of nutrient deprivation with Hank’s Balanced Salt Solution (HBSS) (Sigma Aldrich) for 2 h at 37 °C. After treatment, cells were collected and prepared for experimental procedures described below.

### 2.9. LC3-II Immunoprecipitates

SH-SY5Y cells transfected with an empty construct (CTRL) and stably transfected SH-SY5Y-FLAG-NGB cells untreated and treated with HBSS for 2 h at 37 °C were lysed in lysis buffer as reported in the section for autophagic analysis. The lysates were mixed with protein G-acrylic beads (Sigma-Aldrich) for 2 h at 4 °C and washed extensively. After centrifugation (500× *g* for 1 min), the supernatant was immunoprecipitated with anti-LC3-II pAb (Abcepta, San Diego, CA, USA) plus protein G-acrylic beads. A rabbit IgG isotypic control (Sigma, I5006) was used. The immunoprecipitates were checked by immunoblotting analysis, using anti-LC3-II mAb (Abcam), anti-LAMP1 mAb (Santa Cruz Biotechnology, Dallas, TX, USA) and for the detection NGB using anti-NGB pAb (Santa Cruz Biotechnology).

### 2.10. Statistical Analysis

All statistical analyses were performed by GraphPad Prism software Inc. (version 7, GraphPad Prism Software, San Diego, CA, USA). All data reported were verified in at least 3 different experiments performed in duplicate and reported as mean ± standard deviation (SD) or standard error of the mean (SEM). The *p*-values for all graphs were generated using Student’s *t*-test as reported in the figure legends; * *p* < 0.05, ** *p* < 0.005, *** *p* < 0.001, **** *p* < 0.0001.

## 3. Results

### 3.1. Proteomic Analysis following Genetic Overexpression of NGB in Neuroblastoma SH-SY5Y Cells

**Figure 2:**
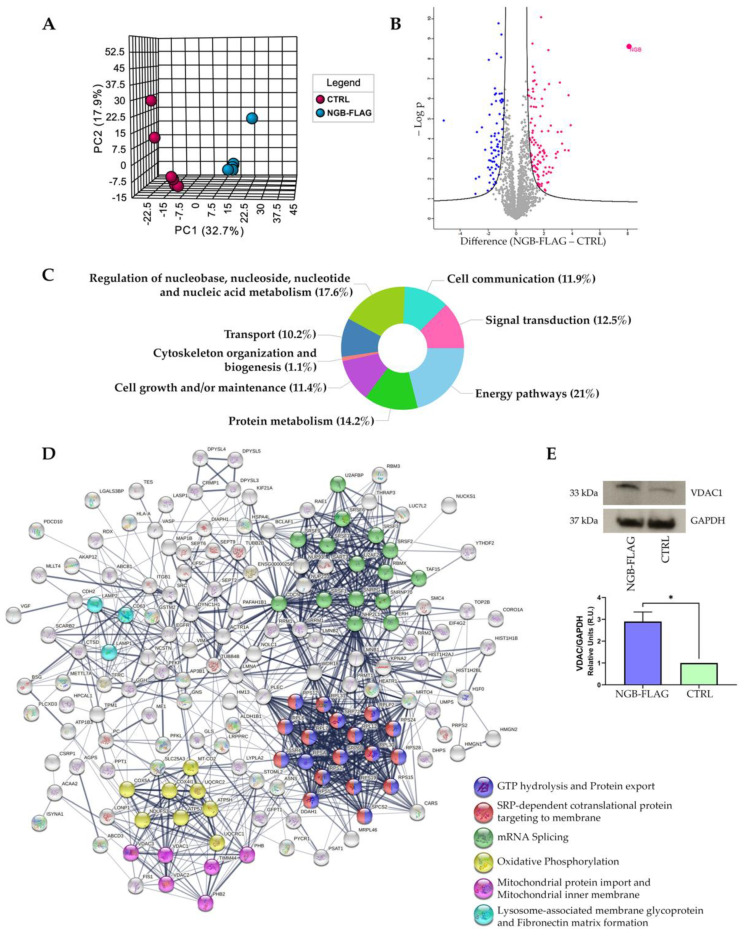
Proteomic analysis following genetic overexpression of NGB in neuroblastoma SH-SY5Y cells. (**A**) The PCA plot shows a good statistical segregation of the two analyzed groups, with principal component (PC)1 = 32.7% and PC2 = 17.9%. (**B**) The volcano plot shows the distribution of the global proteome in NGB-FLAG cells according to the levels of protein Difference (*x*-axis) and statistical significance (*y*-axis). Blue and pink dots represent the statistically significant down- and upregulated proteins, respectively. NGB is visualized in the upper right corner of the graph as the most upregulated protein. Bioinformatics enrichment analysis in NGB-FLAG cells of the (**C**) biological processes and (**D**) PPI clusters obtained using FunRich and STRING software, respectively. (**E**) Validation of proteomic results. The dysregulation of the voltage-dependent anion-selective channel protein 1 (VDAC1) was validated by Western blot. The upper panel shows representative X-ray film scans for the protein and its loading control (GAPDH). The panel at the bottom reports the densitometric mean ± SEM of three immunoblotting replicates with a significant two-tailed *t*-test *p*-value for NGB-FLAG samples with respect to CTRL; * *p* < 0.05.

We preliminarily investigated the effects of *NGB* gene overexpression, stably transfected in a cellular model of human neuroblastoma cells (SH-SY5Y), in the form of a FLAG-tagged protein. Several cellular clones expressing NGB-FLAG were obtained, from which clone 10 was selected on the basis of immunoblotting analysis (Supplementary Figure S1). Precisely, the proteome of NGB-overexpressing cells (NGB-FLAG) was analyzed in comparison to control cells transfected with an empty construct (CTRL) by label-free quantitative (LFQ) proteomics. The PCA revealed a good segregation of the two analyzed groups of samples (Figure 2A). In total, proteome analysis led to the quantification of 1654 out of 2580 identified proteins. Of these, 178 differential proteins (107 up- and 71 down-regulated) were selected on the basis of both relative abundance (Difference) and statistical significance (FDR) levels, then graphed in a volcano plot (Figure 2B). Among these proteins, NGB was uniquely identified in the NGB-FLAG samples and not in the CTRL, as expected, considering the low basal levels of endogenous NGB in SH-SY5Y cells, thus confirming the data obtained by immunoblotting detection. Then, the differential proteome of SH-SY5Y cells was subjected to bioinformatics enrichment analysis to highlight the main cellular pathways and processes enriched by NGB overexpression. The analysis of the biological processes highlighted the most represented terms (Figure 2C), some of which are known to be related to NGB function, such as transport, cytoskeleton organization and energy pathways [43]. Furthermore, the cluster enrichment analysis performed by STRING showed the formation of six major clusters, as reported in the protein–protein interaction (PPI) network in Figure 2D. Interestingly, NGB overexpression may positively regulate the expression of proteins involved in oxidative phosphorylation, mitochondrial transport and lysosomal pathways, which were all upregulated, as revealed by the proteomic analysis. Details of the differential proteins included in these three clusters are reported in Table 2. Among them, one protein of the differential dataset belonging to the cluster of the mitochondrial protein import, the voltage-dependent anion-selective channel protein 1 (VDAC1), a subunit of mitochondrial complex III and cytochrome c1, was chosen and tested as target of technical validation of the proteomic LFQ experiment (Figure 2E). Accordingly, immunoblotting analysis confirmed the increased relative abundance of VDAC1 in NGB-FLAG cells as well as revealed by LFQ analysis (Difference = 1.7).

These observations have provided us with strong indications on the impacted processes to investigate, dependent on overexpressed NGB in neuroblastoma cells. In particular, we decided to perform functional validation of the data retrieved from the LFQ experiment by focusing on two main aspects. First, we explored the effects of the alteration of proteins involved in the oxidative phosphorylation and mitochondrial transport on the whole energy metabolism of SH-SY5Y cells, i.e., VDAC1, considering the possibility for NGB to directly modulate the permeability of the outer mitochondrial membrane through the VDAC1 interaction [16]. Next, we evaluated the specific involvement of NGB in the autolysosome formation during autophagic process by Western blot analysis, since NGB overexpression leads to an increased level of LAMP1 (Lysosome-associated membrane glycoprotein 1).

### 3.2. Effect of NGB Overexpression on the Energy Metabolism in Neuroblastoma SH-SY5Y Cells

To evaluate the differences in bioenergetic metabolism between SH-SY5Y transfected with an empty construct (CTRL) and SH-SY5Y-NGB-FLAG cells, we measured the key parameters of cellular bioenergetics through Seahorse XF technology: oxygen consumption rate (OCR) that allows an estimation of mitochondrial ATP production, and the extracellular acidification rate (ECAR; quantification of glycolytic activity through changes in pH by lactate production), that allows the estimation of ATP produced by glycolysis.

**Figure 3:**
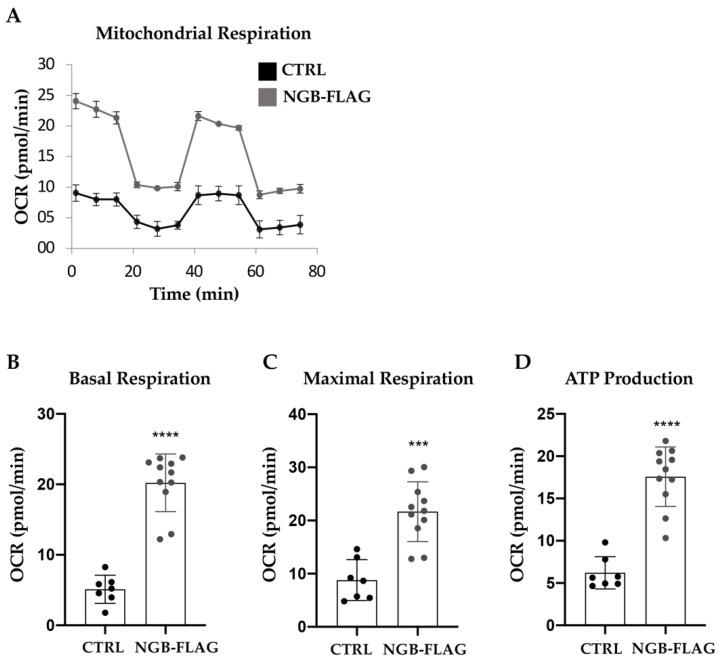
NGB overexpression enhances bioenergetic metabolism of SH-SY5Y cells. (**A**) Mitochondrial stress test profile measuring oxygen consumption rate (OCR) in cells SH-SY5Y and SH-SY5Y stably transfected for expression of neuroglobin. One representative experiment out of four and with each sample in octuplicate, is shown. (**B**–**D**) Individual parameters for basal respiration, ATP production and maximal respiration. Each data point represents an OCR measurement. Data is shown as mean ± SD. Values significantly different from the relative control (SH-SY5Y) are indicated with *** *p* < 0.001, **** *p* < 0.0001.

The overexpression of NGB globally enhanced bioenergetic metabolism of SH-SY5Y-NGB-FLAG cells respect to control cells (Figure 3A). In particular, NGB improved oxidative metabolism increasing respectively basal oxygen consumption (OCR), oxygen consumption uncoupled to ATP production (maximal respiration) and oxygen consumption coupled to ATP synthesis (ATP production); this latter represents an indirect measurement of total cellular ATP production (Figure 3B–D).

**Figure 4:**
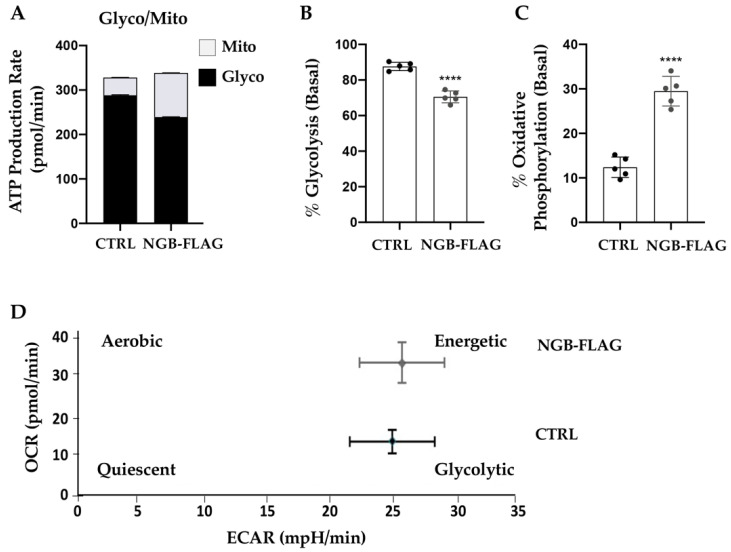
NGB overexpression improves the mitochondrial ATP production and induces a concomitant decrease of the glycolytic ATP in SH-SY5Y cells. (**A**) A comparison of mitochondrial ATP (mitoATP) production rate and glycolytic ATP (glycoATP) production rate between the cells SH-SY5Y and SH-S5Y stably transfected for expression of neuroglobin. (**B**) Mitochondrial OxPhos-linked production of ATP, estimated from the OCR corrected for the P/O ratio. (**C**) Glycolytic production of ATP, estimated from the ECAR following conversion of the pH changes in the absolute amount of H+ released and correction from the CO_2_ release. MitoATP and GlycoATP are shown as stacked bars and are the means ± SEM of five biological replicates; **** *p* < 0.0001. (**D**) Energy map of SH-SY5Y and stably transfected for expression of neuroglobin by graphing baseline OCR versus baseline extracellular acidification rate (ECAR) values.

The ATP assay, performed on SH-SY5Y-NGB-FLAG cells and control cell lines, allowed to discriminate between ATP produced by glycolysis and ATP produced by oxidative phosphorylation. According, to the previous results, NGB overexpression favored mitochondrial ATP production reducing the ATP produced by glycolysis in SH-SY5-NGB-FLAG cells (Figure 4A–C). The energy map summarized the bioenergetic changes induced by NGB overexpression in SH-SY5Y cells, showing the transfected cell line positioned in an aerobic/energetic area of the map, with respect to the control cell line positioned in a glycolytic area (Figure 4D).

### 3.3. NGB Overexpression Increases NRF1 mRNA and Mitochondrial DNA Levels in Neuroblastoma SH-SY5Y Cells

**Figure 5:**
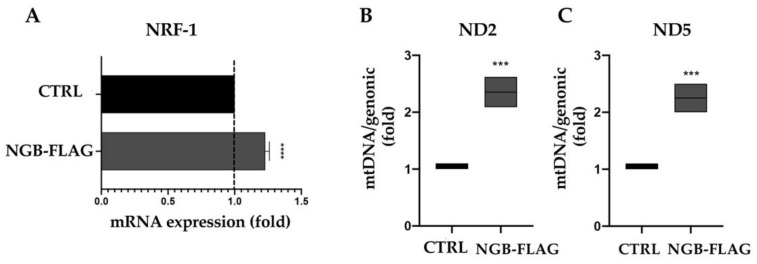
NGB overexpression increases NRF1 mRNA and mitochondrial DNA levels in SH-SY5Y cells. (**A**) Expression of mRNAs coding for NRF1 in the SH-SY5Y cells transfected with an empty vector (CTRL) and SH-SY5Y stably transfected for expression of neuroglobin. (**B**,**C**) RT-qPCR quantification of ND2 and ND5 genes encoded by mitochondrial DNA in the CTRL and SH-SY5Y stably transfected for expression of neuroglobin. The control cells were arbitrary set at 1. Data are presented as mean ± SEM, *** *p* < 0.001 **** *p* < 0.0001, unpaired Student’s *t* test, RT-qPCR *n* = 4.

To support our findings on the role of NGB overexpression in increasing energetic metabolism, we analyzed NRF1 mRNA. Since nuclear respiratory factor 1 (NRF1) is a transcription factor which activates the expression of some key metabolic genes regulating cellular growth and nuclear genes required for respiration, heme biosynthesis and mitochondrial DNA transcription and replication. Our aim was to analyze NRF1 mRNA expression levels in SH-SY5Y cells. As shown in Figure 5A, NRF1 mRNA levels displayed up-regulation under NGB overexpression in SH-SY5Y cells, as compared to control cells. Simultaneously, levels of mitochondrial DNA were significantly higher in SHSY5Y-NGB FLAG cells as compared to control cells, as revealed by the mt DNA/genomic DNA ratio (Figure 5B,C).

### 3.4. Effect of NGB Overexpression on Autophagy Induction in Neuroblastoma SH-SY5Y Cells

**Figure 6:**
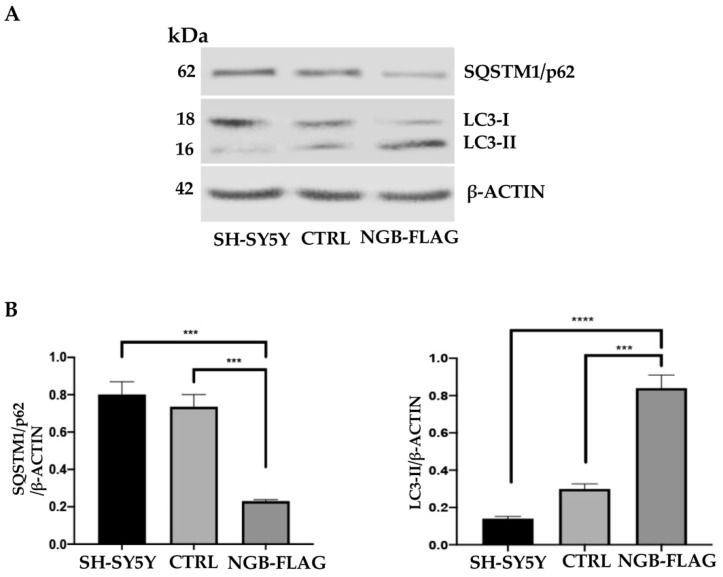
Effect of NGB overexpression on autophagy induction in SH-SY5Y cells. (**A**) SH-SY5Y cells, SH-SY5Y cells transfected with an empty construct (CTRL) and stably transfected SH-SY5Y FLAG-NGB cells were lysed in lysis buffer, subjected to 15% SDS-PAGE and analyzed by Western blot using anti-LC3 polyclonal antibody or rabbit anti-SQSTM1 mAb. Loading control was evaluated using anti-ACTB mAb. A representative experiment among 3 is shown. (**B**) Bar graph on the right shows densitometric analysis. Results represent the mean ± SD from 3 independent experiments. *** *p* < 0.001, **** *p* < 0.0001.

Since a bidirectional relationship between autophagy and mitochondrial metabolism was reported in cancer cells, in which mitochondria regulate the supply of free fatty acid by regulating the formation of autophagosomes [57], we investigated the effect of NGB overexpression on autophagy induction. Endogenous LC3-II and SQSTM1/p62 levels were measured by immunoblotting, using anti-MAP1LC3/LC3 (microtubule associated protein 1 light chain 3) or anti-SQSTM1/p62 (sequestosome1) antibodies (Figure 6). All these proteins were modulated by NGB overexpression, indicating a cellular condition that is more prone to autophagy process. Indeed, Western blot analysis showed an increase of LC3-II together with a significant decrease of SQSTM1/p62 in SH-SY5Y-NGB-FLAG as compared to the control cells, as also confirmed by densitometric analysis. Similar findings were obtained in neuroblastoma SK-N-BE2-NGB FLAG cells (Supplementary Figure S2).

### 3.5. Effect of NGB Overexpression on the Lysosome Compartments of Neuroblastoma SH-SY5Y Cells

**Figure 7:**
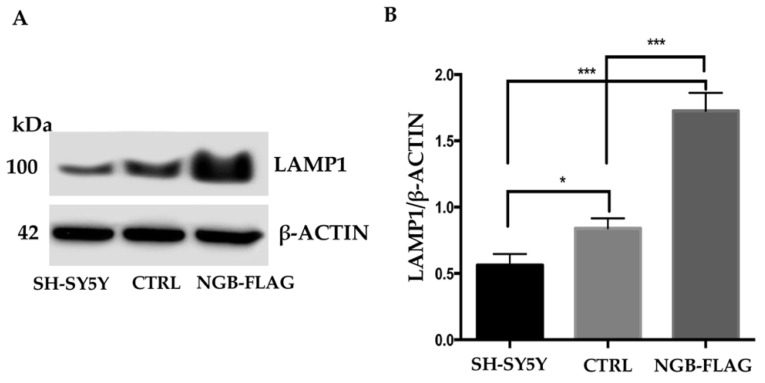
Effect of NGB overexpression on LAMP1 expression in SH-SY5Y cells. (**A**) SH-SY5Y cells, SH-SY5Y cells transfected with an empty construct (CTRL) and stably transfected SH-SY5Y-NGB-FLAG cells were lysed in lysis buffer, subjected to 7.5% SDS-PAGE and analyzed by Western blot using anti-LAMP1 mAb. Loading control was evaluated using anti-ACTB mAb. A representative experiment among 3 is shown. (**B**) Bar graph shows densitometric analysis. Results represent the mean ± SD from 3 independent experiments. * *p* < 0.05, *** *p* < 0.001.

To confirm a possible link between NGB and autophagy process, we investigated the role of NGB in the formation of autolysosome. We first validated the results obtained by LFQ proteomic analysis, which revealed that NGB overexpression leads to an increased expression of LAMP1 (log_2_ Difference of 2.3, Table 2), a structural protein of lysosome/late endosomes, used as a marker for autophagy flux confirmation [58]. As expected, Western blot analysis confirmed a significant increase of LAMP1 in NGB-overexpressing cells in comparison with both CTRL and wild type SH-SY5Y cells, as revealed by densitometric analysis (Figure 7A,B).

### 3.6. NGB Associates with LC3-II during Autolysosome Formation in Neuroblastoma SH-SY5Y-NGB-FLAG Cells

**Figure 8:**
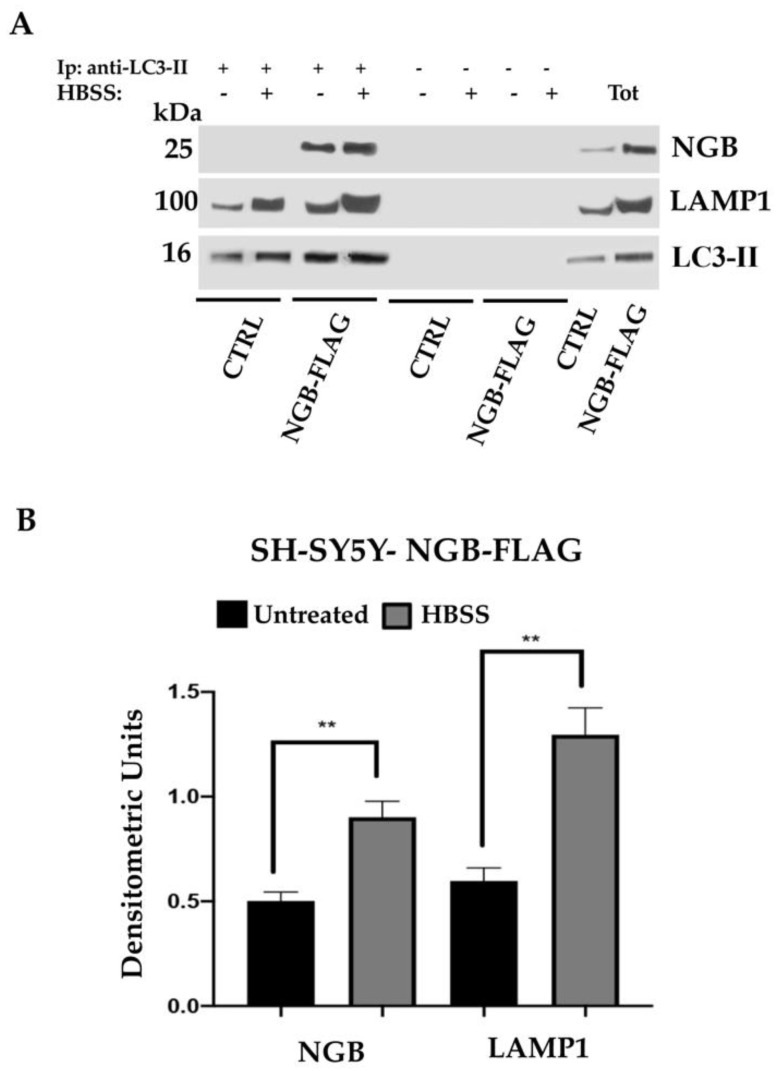
NGB associates with LC3-II during autolysosome formation in SH-SY5Y cells. (**A**) SH-SY5Y cells transfected with an empty construct (CTRL) and stably transfected SH-SY5Y-NGB-FLAG cells untreated or treated with HBSS for 2 h at 37 °C, were lysed in lysis buffer, followed by immunoprecipitation with rabbit anti-LC3-II. A rabbit IgG isotypic control (IpCtr) was employed. The immunoprecipitates were checked by Western blot analysis, using anti-LC3-II mAb, anti-LAMP1 mAb and anti-NGB pAb. A representative experiment among 3 is shown. (**B**) Bar graph shows densitometric analysis. Results represent the mean ± SD from 3 independent experiments. ** *p* < 0.005.

We then evaluated the role of NGB in the autolysosome formation. In agreement with the evidence that LC3-II colocalizes with LAMP1 during autophagy [59], we analyzed by coimmunoprecipitation experiments the possible association of NGB with LC3-II-LAMP1 complex following autophagy induction (Figure 8A,B). After triggering with HBSS, a significant proportion of NGB became associated with LC3-II, which was more evident in stably transfected SH-SY5Y-NGB-FLAG cells treated with HBSS for 2 h in comparison with untreated cells. In the same LC3-II immunoprecipitates, we also evaluated the association with LAMP1 and observed a positive band of coimmunoprecipitation which was more evident in cells stimulated with HBSS for 2 h. No bands were detected in control immunoprecipitation experiments with an IgG having irrelevant specificity. LC3-II immunoprecipitation was verified by Western blot. Altogether, these results clearly suggest that the induction of autophagy could trigger a molecular interaction between NGB and a complex of two key molecules, such as LC3-II and LAMP1, involved in autolysosome formation.

## 4. Discussion

In this study, we show the role for NGB in enhancing mitochondrial energy metabolism, as well as its involvement of NGB in autolysosome formation. In a previous study, we demonstrated a neuroprotective activity of overexpressed NGB associated specifically with mitochondrial raft-like microdomains in a human neuroblastoma cell line triggered with the neurotoxin MPP+ [16]. In the current work, we propose a possible mechanism through which NGB may exert its protective role. Furthermore, we used for the first time a proteomic approach with the aim of identifying the specific profile following the overexpression of the NGB. This approach revealed several differentially regulated proteins, including those proteins involved in oxidative phosphorylation and integral mitochondrial proteins linked to energy metabolism. These findings suggested a direct or indirect involvement of NGB in a variety of mitochondrial pathways. In particular, the analysis of mitochondrial metabolism clearly demonstrated that overexpression of NGB increases mitochondrial ATP production and induces a concomitant decrease of the glycolytic ATP in SH-SY5Y-NGB-FLAG cells. The energy map confirmed the increase in the oxidative capacity of the overexpressing NGB cells, with a decrease in the glycolytic function. Indeed, overexpression of NGB globally enhanced bioenergetic metabolism, increasing basal oxygen consumption (OCR), oxygen consumption uncoupled to ATP production (maximal respiration) as well as oxygen consumption coupled to ATP synthesis. To support the role of NGB overexpression in increasing energy metabolism, we analyzed NRF1 mRNA, since it encodes a protein that functions as a key transcription factor required for respiration, heme biosynthesis and mitochondrial DNA transcription. The increase of NRF1 mRNA and mitochondrial DNA levels clearly indicated the increase in the mitochondrial biogenesis in NGB overexpressing cells. Several evidence highlighted that stressing conditions induce a re-localization of endogenous human NGB to mitochondria, where it displays a pivotal redox-dependent protective role against neurodegeneration by preventing neuronal apoptosis and mitochondrial damage [16,60]. Furthermore, Bosc et al. [57] suggested a functional link between autophagy, lipid metabolism and oxidative phosphorylation: autophagy participates in lipid catabolism to support oxidative phosphorylation, showing that autophagy occurring at this specific contact sites between the endoplasmic reticulum (ER) and mitochondria regulated fatty acid availability for oxidative phosphorylation to maintain mitochondrial energy metabolism in cancer cells. In fact, in a previous paper we demonstrated that upon cell oxidative stress, NGB re-localizes to mitochondria at level of mitochondrial raft-like microdomains, where it may interact with lipid rafts components [16]. These microdomains represent preferential sites on the mitochondrial membrane where some key reactions can be catalyzed, thus contributing to cell apoptosis and autophagy [61,62,63,64,65,66]. In particular, our recent papers demonstrated that mitochondrial raft-like microdomains, at the contact site between mitochondria and ER, may participate to the formation of autophagosome during autophagy process [66,67,68].

Autophagy is an intracellular degradation system that supplies cytoplasmic materials to the lysosome or vacuole. In all kinds of stress conditions as well as in pathological conditions, potentiation of autophagy is known to play a role as a protective mechanism. Considering that NGB can act in a protective way in various pathological circumstances, including oncological pathologies, in the present study we verified whether this globin could represent a critical regulator of mitochondrial metabolism and autophagy. Although other authors have hypothesized an extraneousness of NGB in the autophagic machinery [29], our results clearly demonstrated a role of overexpressed NGB, in fact HBSS-induced autophagy triggered a molecular interaction between NGB and a two key molecules complex, such as LC3-II and LAMP1, involved in autolysosome formation. Therefore, the involvement of NGB with autophagic machinery could represent one of the pathways to prevent cell death associated with stressing conditions. Autophagy has been found frequently activated in cancer and inevitably associated with metabolism. In fact, from this point of view, autophagy is also to be considered as a crucial process capable of promoting cancer survival under metabolic and genotoxic stress, endowing cancer cells with resistance to treatment, even if some support an ambivalent role of autophagy in cancer [29,69,70,71]. In this regard, some papers have suggested an involvement of autophagy in resistance to apoptosis and in some cases autophagy and apoptosis have been found to be inversely correlated [72,73,74]. The antiapoptotic role of NGB against oxidative stress, as well as other hypothetical functions of this globin, seems to be linked to the set of factors of expression, localization and interaction of NGB with other proteins [15,75].

In this concern, our data suggest a potential metabolic compensatory mechanism regulated by autophagy activation in which NGB plays a pivotal role. Cells can limit mitochondrial damage thanks to various surveillance strategies [76,77], such as mitochondrial biogenesis, dynamic (fusion/fission) and selective mitochondrial autophagy. Mitochondria therefore have different systems of proteostasis; the failure of one of these mechanisms always opens the possibility of an alternative way to remove damaged mitochondria [31,78,79].

## 5. Conclusions

In the current paper, we highlighted the active participation of NGB in several cellular processes that seem to be upregulated in response to NGB overexpression. Overall, our hypothesis is that NGB plays a role in orchestrating stress adaptation in both neural and non-neural cells as well as in cancer. This role is highlighted not only by the participation of NGB in the autophagic flow, but also in the activation of oxidative phosphorylation and in the increase of mitochondrial mass.

## Figures and Tables

**Table 1:** Target genes and oligonucleotide sequences.

Target	Forward Sequence	Reverse Sequence
ND5	5′-AGCATTCGGAAGCATCTTTG-3′	3′-TCGGATGTCTTGTTCGTCTG-5′
ND2	5′-CTACCGCATTCCTACTACTCAACTT-3′	3′-GCTTGTTTCAGGTGCGAGAT-5′
NRF1	5′-CAGCCGCTCTGAGAACTTCA- 3′	3′-CGGTGTAAGTAGCCACATGGA-5′
IL2	5′TAGGCCACAGAATTGAAAGATCT- 3′	3′GTAGGTGGAAATTCTAGCATCATCC-5′

**Table 2:** Description of the quantitative changes related to the proteins belonging to the clusters identified by STRING analysis in NGB-overexpressing SH-SY5Y cells.

UniProt ID	Gene Name	Protein Description	Difference	Cluster
P13073	*COX4I1*	Cytochrome c oxidase subunit 4 isoform 1, mitochondrial	2.3	Oxidative Phosphorylation
P56134	*ATP5J2*	ATP synthase subunit f, mitochondrial	2.2	
P31930	*UQCRC1*	Cytochrome b-c1 complex subunit 1, mitochondrial	2.0	
O75947	*ATP5H*	ATP synthase subunit d, mitochondrial	1.9	
P22695	*UQCRC2*	Cytochrome b-c1 complex subunit 2, mitochondrial	1.7	
P20674	*COX5A*	Cytochrome c oxidase subunit 5A, mitochondrial	1.6	
O75489	*NDUFS3*	NADH dehydrogenase [ubiquinone] iron-sulfur protein 3, mitochondrial	1.4	
P00403	*MT-CO2*	Cytochrome c oxidase subunit 2	1.3	
Q99623	*PHB2*	Prohibitin-2	1.8	Mitochondrial protein import, and integral component of mitochondrial inner membrane
P35232	*PHB*	Prohibitin	1.7	
P21796	*VDAC1*	Voltage-dependent anion-selective channel protein 1	1.7	
Q9Y277	*VDAC3*	Voltage-dependent anion-selective channel protein 3	1.5	
O43615	*TIMM44*	Mitochondrial import inner membrane translocase subunit TIM44	1.5	
P45880	*VDAC2*	Voltage-dependent anion-selective channel protein 2	1.4	
P08962	*CD63*	CD63 antigen	3.9	Lysosome-associated membrane glycoprotein, and Fibronectin matrix formation
P11279	*LAMP1*	Lysosome-associated membrane glycoprotein 1	2.3	
P13473	*LAMP2*	Lysosome-associated membrane glycoprotein 2	2.0	

Proteins were ordered for each cluster according to decrescent values of Difference.

## Data Availability

The mass spectrometry proteomics data have been deposited to the ProteomeXchange Consortium via the PRIDE partner repository with the dataset identifier PXD029012.

## Supplementary Materials

The following are available online at https://www.mdpi.com/article/10.3390/cells10123394/s1, Figure S1: Immunoblotting analysis of the cellular clones expressing NGB-FLAG; Figure S2: Effect of NGB overexpression on autophagy induction in SK-N-BE2 cells.

## References

[1] Burmester T., Weich B., Reinhardt S., Hankeln T. (2000). A vertebrate globin expressed in the brain. Nature.

[2] Burmester T., Hankeln T. (2009). What is the function of neuroglobin?. J. Exp. Biol..

[3] Ascenzi P., Gustincich S., Marino M. (2014). Mammalian nerve globins in search of functions. IUBMB Life.

[4] Yu Z., Xu J., Liu N., Wang Y., Li X., Pallast S., van Leyen K., Wang X. (2012). Mitochondrial distribution of neuroglobin and its response to oxygen-glucose deprivation in primary-cultured mouse cortical neurons. Neuroscience.

[5] Yu Z., Poppe J.L., Wang X. (2013). Mitochondrial mechanisms of neuroglobin’s neuroprotection. Oxidative Med. Cell. Longev..

[6] Raychaudhuri S., Skommer J., Henty K., Birch N., Brittain T. (2010). Neuroglobin protects nerve cells from apoptosis by inhibiting the intrinsic pathway of cell death. Apoptosis.

[7] Fordel E., Thijs L., Moens L., Dewilde S. (2007). Neuroglobin and cytoglobin expression in mice. Evidence for a correlation with reactive oxygen species scavenging. FEBS J..

[8] Schmidt-Kastner R., Haberkamp M., Schmitz C., Hankeln T., Burmester T. (2006). Neuroglobin mRNA expression after transient global brain ischemia and prolonged hypoxia in cell culture. Brain Res..

[9] Ye S.Q., Zhou X.Y., Lai X.J., Zheng L., Chen X.Q. (2009). Silencing neuroglobin enhances neuronal vulnerability to oxidative injury by down-regulating 14-3-3gamma. Acta Pharmacol. Sin..

[10] Shao G., Gong K.R., Li J., Xu X.J., Gao C.Y., Zeng X.Z., Lu G.W., Huo X. (2009). Antihypoxic effects of neuroglobin in hypoxia-preconditioned mice and SH-SY5Y cells. Neurosignals.

[11] Hundahl C.A., Allen G.C., Hannibal J., Kjaer K., Rehfeld J.F., Dewilde S., Nyengaard J.R., Kelsen J., Hay-Schmidt A. (2010). Anatomical characterization of cytoglobin and neuroglobin mRNA and protein expression in the mouse brain. Brain Res..

[12] Bentmann A., Schmidt M., Reuss S., Wolfrum U., Hankeln T., Burmester T. (2005). Divergent distribution in vascular and avascular mammalian retinae links neuroglobin to cellular respiration. J. Biol. Chem..

[13] De Marinis E., Fiocchetti M., Acconcia F., Ascenzi P., Marino M. (2013). Neuroglobin upregulation induced by 17β-estradiol sequesters cytocrome c in the mitochondria preventing H_2_O_2_-induced apoptosis of neuroblastoma cells. Cell Death Dis..

[14] Fiocchetti M., Cipolletti M., Brandi V., Polticelli F., Ascenzi P. (2017). Neuroglobin and friends. J. Mol. Recognit..

[15] Fiocchetti M., Cipolletti M., Leone S., Naldini A., Carraro F., Giordano D., Verde C., Ascenzi P., Marino M. (2016). Neuroglobin in Breast Cancer Cells: Effect of Hypoxia and Oxidative Stress on Protein Level, Localization, and Anti-Apoptotic Function. PLoS ONE.

[16] Garofalo T., Ferri A., Sorice M., Azmoon P., Grasso M., Mattei V., Capozzi A., Manganelli V., Misasi R. (2018). Neuroglobin overexpression plays a pivotal role in neuroprotection through mitochondrial raft-like microdomains in neuroblastoma SK-N-BE2 cells. Mol. Cell. Neurosci..

[17] Schieber M., Chandel N.S. (2014). ROS function in redox signaling and oxidative stress. Curr. Biol..

[18] Solar Fernandez V., Cipolletti M., Ascenzi P., Marino M., Fiocchetti M. (2020). Neuroglobin as Key Mediator in the 17β-Estradiol-Induced Antioxidant Cell Response to Oxidative Stress. Antioxid. Redox Signal..

[19] Li R.C., Morris M.W., Lee S.K., Pouranfar F., Wang Y., Gozal D. (2008). Neuroglobin protects PC12 cells against oxidative stress. Brain Res..

[20] Khan A.A., Wang Y., Sun Y., Mao X.O., Xie L., Miles E., Graboski J., Chen S., Ellerby L.M., Jin K. (2006). Neuroglobin-overexpressing transgenic mice are resistant to cerebral and myocardial ischemia. Proc. Natl. Acad. Sci. USA.

[21] Liu J., Yu Z., Guo S., Lee S.R., Xing C., Zhang C., Gao Y., Nicholls D.G., Lo E.H., Wang X. (2009). Effects of neuroglobin overexpression on mitochondrial function and oxidative stress following hypoxia/reoxygenation in cultured neurons. J. Neurosci. Res..

[22] Watanabe S., Takahashi N., Uchida H., Wakasugi K. (2012). Human neuroglobin functions as an oxidative stress-responsive sensor for neuroprotection. J. Biol. Chem..

[23] De Marinis E., Ascenzi P., Pellegrini M., Galluzzo P., Bulzomi P., Arevalo M.A., Garcia-Segura L.M., Marino M. (2010). 17β-estradiol a new modulator of neuroglobin levels in neurons: Role in neuroprotection against H_2_O_2_-induced toxicity. Neurosignals.

[24] Lan W.B., Lin J.H., Chen X.W., Wu C.Y., Zhong G.X., Zhang L.Q., Lin W.P., Liu W.N., Li X., Lin J.L. (2014). Overexpressing neuroglobin improves functional recovery by inhibiting neuronal apoptosis after spinal cord injury. Brain Res..

[25] Fiocchetti M., De Marinis E., Ascenzi P., Marino M. (2013). Neuroglobin and neuronal cell survival. Biochim. Biophys. Acta (BBA)-Proteins Proteom..

[26] Yu Z., Liu N., Liu J., Yang K., Wang X. (2012). Neuroglobin, a Novel Target for Endogenous Neuroprotection against Stroke and Neurodegenerative Disorders. Int. J. Mol. Sci..

[27] Hara T., Nakamura K., Matsui M., Yamamoto A., Nakahara Y., Suzuki-Migishima R., Yokoyama M., Mishima K., Saito I., Okano H. (2006). Suppression of basal autophagy in neural cells causes neurodegenerative disease in mice. Nature.

[28] Dadakhujaev S., Noh H.S., Jung E.J., Cha J.Y., Baek S.M., Ha J.H., Kim D.R. (2010). Autophagy protects the rotenone-induced cell death in alpha-synuclein overexpressing SH-SY5Y cells. Neurosci. Lett..

[29] Fiocchetti M., Cipolletti M., Marino M. (2017). Compensatory role of Neuroglobin in nervous and non-nervous cancer cells in response to the nutrient deprivation. PLoS ONE.

[30] Sheng Z.H., Cai Q. (2012). Mitochondrial transport in neurons: Impact on synaptic homeostasis and neurodegeneration. Nat. Rev. Neurosci..

[31] Cai Q., Tammineni P. (2016). Alterations in Mitochondrial Quality Control in Alzheimer’s Disease. Front. Cell. Neurosci..

[32] Lin M.T., Beal M.F. (2006). Mitochondrial dysfunction and oxidative stress in neurodegenerative diseases. Nature.

[33] Zhunina O.A., Yabbarov N.G., Grechko A.V., Starodubova A.V., Ivanova E., Nikiforov N.G., Orekhov A.N. (2021). The Role of Mitochondrial Dysfunction in Vascular Disease, Tumorigenesis, and Diabetes. Front. Mol. Biosci..

[34] Malpartida A.B., Williamson M., Narendra D.P., Wade-Martins R., Ryan B.J. (2021). Mitochondrial Dysfunction and Mitophagy in Parkinson’s Disease: From Mechanism to Therapy. Trends Biochem. Sci..

[35] Elkamhawy A., Lee J., Park B.G., Park I., Pae A.N., Roh E.J. (2014). Novel quinazoline-urea analogues as modulators for Aβ-induced mitochondrial dysfunction: Design, synthesis, and molecular docking study. Eur. J. Med. Chem..

[36] Elkamhawy A., Park J.E., Hassan A.H.E., Pae A.N., Lee J., Paik S., Park B.G., Roh E.J. (2018). Pyrazinyl ureas revisited: 1-(3-(Benzyloxy)pyrazin-2-yl)-3-(3,4-dichlorophenyl)urea, a new blocker of Aβ-induced mPTP opening for Alzheimer’s disease. Eur. J. Med. Chem..

[37] Ji C.H., Kwon Y.T. (2017). Crosstalk and Interplay between the Ubiquitin-Proteasome System and Autophagy. Mol. Cells.

[38] Elkamhawy A., Kim H.J., Elsherbeny M.H., Paik S., Park J.H., Gotina L., Abdellattif M.H., Gouda N.A., Cho J., Lee K. (2021). Discovery of 3,4-dichloro-N-(1H-indol-5-yl)benzamide: A highly potent, selective, and competitive hMAO-B inhibitor with high BBB permeability profile and neuroprotective action. Bioorganic Chem..

[39] Elkamhawy A., Woo J., Gouda N.A., Kim J., Nada H., Roh E.J., Park K.D., Cho J., Lee K. (2021). Melatonin Analogues Potently Inhibit MAO-B and Protect PC12 Cells against Oxidative Stress. Antioxidants.

[40] Elsherbeny M.H., Kim J., Gouda N.A., Gotina L., Cho J., Pae A.N., Lee K., Park K.D., Elkamhawy A., Roh E.J. (2021). Highly Potent, Selective, and Competitive Indole-Based MAO-B Inhibitors Protect PC12 Cells against 6-Hydroxydopamine- and Rotenone-Induced Oxidative Stress. Antioxidants.

[41] Strother L., Miles G.B., Holiday A.R., Cheng Y., Doherty G.H. (2021). Long-term culture of SH-SY5Y neuroblastoma cells in the absence of neurotrophins: A novel model of neuronal ageing. J. Neurosci. Methods.

[42] Xu Z., Yang D., Huang X., Huang H. (2021). Astragaloside IV Protects 6-Hydroxydopamine-Induced SH-SY5Y Cell Model of Parkinson’s Disease via Activating the JAK2/STAT3 Pathway. Front. Neurosci..

[43] Costanzo M., Fiocchetti M., Ascenzi P., Marino M., Caterino M., Ruoppolo M. (2021). Proteomic and Bioinformatic Investigation of Altered Pathways in Neuroglobin-Deficient Breast Cancer Cells. Molecules.

[44] Cozzolino M., Amori I., Pesaresi M.G., Ferri A., Nencini M., Carrì M.T. (2008). Cysteine 111 affects aggregation and cytotoxicity of mutant Cu,Zn-superoxide dismutase associated with familial amyotrophic lateral sclerosis. J. Biol. Chem..

[45] De Pasquale V., Costanzo M., Siciliano R.A., Mazzeo M.F., Pistorio V., Bianchi L., Marchese E., Ruoppolo M., Pavone L.M., Caterino M. (2020). Proteomic Analysis of Mucopolysaccharidosis IIIB Mouse Brain. Biomolecules.

[46] De Pasquale V., Caterino M., Costanzo M., Fedele R., Ruoppolo M., Pavone L.M. (2020). Targeted Metabolomic Analysis of a Mucopolysaccharidosis IIIB Mouse Model Reveals an Imbalance of Branched-Chain Amino Acid and Fatty Acid Metabolism. Int. J. Mol. Sci..

[47] Giacco A., Delli Paoli G., Senese R., Cioffi F., Silvestri E., Moreno M., Ruoppolo M., Caterino M., Costanzo M., Lombardi A. (2019). The saturation degree of fatty acids and their derived acylcarnitines determines the direct effect of metabolically active thyroid hormones on insulin sensitivity in skeletal muscle cells. FASEB J..

[48] Costanzo M., Cevenini A., Marchese E., Imperlini E., Raia M., Del Vecchio L., Caterino M., Ruoppolo M. (2018). Label-Free Quantitative Proteomics in a Methylmalonyl-CoA Mutase-Silenced Neuroblastoma Cell Line. Int. J. Mol. Sci..

[49] Costanzo M., Caterino M., Cevenini A., Jung V., Chhuon C., Lipecka J., Fedele R., Guerrera I.C., Ruoppolo M. (2020). Proteomics Reveals that Methylmalonyl-CoA Mutase Modulates Cell Architecture and Increases Susceptibility to Stress. Int. J. Mol. Sci..

[50] Melo M.G., Remacle N., Cudré-Cung H.P., Roux C., Poms M., Cudalbu C., Barroso M., Gersting S.W., Feichtinger R.G., Mayr J.A. (2021). The first knock-in rat model for glutaric aciduria type I allows further insights into pathophysiology in brain and periphery. Mol. Genet. Metab..

[51] Costanzo M., Caterino M., Cevenini A., Jung V., Chhuon C., Lipecka J., Fedele R., Guerrera I.C., Ruoppolo M. (2020). Dataset of a comparative proteomics experiment in a methylmalonyl-CoA mutase knockout HEK 293 cell model. Data Brief.

[52] Caterino M., Ruoppolo M., Villani G.R.D., Marchese E., Costanzo M., Sotgiu G., Dore S., Franconi F., Campesi I. (2020). Influence of Sex on Urinary Organic Acids: A Cross-Sectional Study in Children. Int. J. Mol. Sci..

[53] Caterino M., Costanzo M., Fedele R., Cevenini A., Gelzo M., Di Minno A., Andolfo I., Capasso M., Russo R., Annunziata A. (2021). The Serum Metabolome of Moderate and Severe COVID-19 Patients Reflects Possible Liver Alterations Involving Carbon and Nitrogen Metabolism. Int. J. Mol. Sci..

[54] Fonseka P., Pathan M., Chitti S.V., Kang T., Mathivanan S. (2021). FunRich enables enrichment analysis of OMICs datasets. J. Mol. Biol..

[55] Caterino M., Ruoppolo M., Mandola A., Costanzo M., Orrù S., Imperlini E. (2017). Protein-protein interaction networks as a new perspective to evaluate distinct functional roles of voltage-dependent anion channel isoforms. Mol. Biosyst..

[56] Miller F.J., Rosenfeldt F.L., Zhang C., Linnane A.W., Nagley P. (2003). Precise determination of mitochondrial DNA copy number in human skeletal and cardiac muscle by a PCR-based assay: Lack of change of copy number with age. Nucleic Acids Res..

[57] Bosc C., Broin N., Fanjul M., Saland E., Farge T., Courdy C., Batut A., Masoud R., Larrue C., Skuli S. (2020). Autophagy regulates fatty acid availability for oxidative phosphorylation through mitochondria-endoplasmic reticulum contact sites. Nat. Commun..

[58] Klionsky D.J., Abdel-Aziz A.K., Abdelfatah S., Abdellatif M., Abdoli A., Abel S., Abeliovich H., Abildgaard M.H., Abudu Y.P., Acevedo-Arozena A. (2021). Guidelines for the use and interpretation of assays for monitoring autophagy (4th edition). Autophagy.

[59] Furuta N., Yoshimori T., Amano A. (2010). Mediatory molecules that fuse autophagosomes and lysosomes. Autophagy.

[60] Singh S., Zhuo M., Gorgun F.M., Englander E.W. (2013). Overexpressed neuroglobin raises threshold for nitric oxide-induced impairment of mitochondrial respiratory activities and stress signaling in primary cortical neurons. Nitric Oxide.

[61] Barbat C., Trucy M., Sorice M., Garofalo T., Manganelli V., Fischer A., Mazerolles F. (2007). p56lck, LFA-1 and PI3K but not SHP-2 interact with GM1- or GM3-enriched microdomains in a CD4-p56lck association-dependent manner. Biochem. J..

[62] Ciarlo L., Manganelli V., Matarrese P., Garofalo T., Tinari A., Gambardella L., Marconi M., Grasso M., Misasi R., Sorice M. (2012). Raft-like microdomains play a key role in mitochondrial impairment in lymphoid cells from patients with Huntington’s disease. J. Lipid Res..

[63] Sorice M., Mattei V., Tasciotti V., Manganelli V., Garofalo T., Misasi R. (2012). Trafficking of PrPc to mitochondrial raft-like microdomains during cell apoptosis. Prion.

[64] Iessi E., Marconi M., Manganelli V., Sorice M., Malorni W., Garofalo T., Matarrese P. (2020). On the role of sphingolipids in cell survival and death. Int. Rev. Cell. Mol. Biol..

[65] Matarrese P., Garofalo T., Manganelli V., Gambardella L., Marconi M., Grasso M., Tinari A., Misasi R., Malorni W., Sorice M. (2014). Evidence for the involvement of GD3 ganglioside in autophagosome formation and maturation. Autophagy.

[66] Garofalo T., Matarrese P., Manganelli V., Marconi M., Tinari A., Gambardella L., Faggioni A., Misasi R., Sorice M., Malorni W. (2016). Evidence for the involvement of lipid rafts localized at the ER-mitochondria associated membranes in autophagosome formation. Autophagy.

[67] Manganelli V., Capozzi A., Recalchi S., Riitano G., Mattei V., Longo A., Misasi R., Garofalo T., Sorice M. (2021). The Role of Cardiolipin as a Scaffold Mitochondrial Phospholipid in Autophagosome Formation: In Vitro Evidence. Biomolecules.

[68] Manganelli V., Matarrese P., Antonioli M., Gambardella L., Vescovo T., Gretzmeier C., Longo A., Capozzi A., Recalchi S., Riitano G. (2021). Raft-like lipid microdomains drive autophagy initiation via AMBRA1-ERLIN1 molecular association within MAMs. Autophagy.

[69] Jain K., Paranandi K.S., Sridharan S., Basu A. (2013). Autophagy in breast cancer and its implications for therapy. Am. J. Cancer Res..

[70] Karantza-Wadsworth V., White E. (2007). Role of autophagy in breast cancer. Autophagy.

[71] Mathew R., Karantza-Wadsworth V., White E. (2007). Role of autophagy in cancer. Nat. Rev. Cancer.

[72] Mariño G., Niso-Santano M., Baehrecke E.H., Kroemer G. (2014). Self-consumption: The interplay of autophagy and apoptosis. Nat. Rev. Mol. Cell. Biol..

[73] Vomero M., Manganelli V., Barbati C., Colasanti T., Capozzi A., Finucci A., Spinelli F.R., Ceccarelli F., Perricone C., Truglia S. (2019). Reduction of autophagy and increase in apoptosis correlates with a favorable clinical outcome in patients with rheumatoid arthritis treated with anti-TNF drugs. Arthritis Res. Ther..

[74] Manganelli V., Longo A., Mattei V., Recalchi S., Riitano G., Caissutti D., Capozzi A., Sorice M., Misasi R., Garofalo T. (2021). Role of ERLINs in the Control of Cell Fate through Lipid Rafts. Cells.

[75] Fiocchetti M., Solar Fernandez V., Segatto M., Leone S., Cercola P., Massari A., Cavaliere F., Marino M. (2020). Extracellular Neuroglobin as a Stress-Induced Factor Activating Pre-Adaptation Mechanisms against Oxidative Stress and Chemotherapy-Induced Cell Death in Breast Cancer. Cancers.

[76] Roca-Portoles A., Tait S.W.G. (2021). Mitochondrial quality control: From molecule to organelle. Cell. Mol. Life Sci..

[77] Panchal K., Tiwari A.K. (2019). Mitochondrial dynamics, a key executioner in neurodegenerative diseases. Mitochondrion.

[78] Han S., Zhang M., Jeong Y.Y., Margolis D.J., Cai Q. (2021). The role of mitophagy in the regulation of mitochondrial energetic status in neurons. Autophagy.

[79] Sorrentino V., Menzies K.J., Auwerx J. (2018). Repairing Mitochondrial Dysfunction in Disease. Annu. Rev. Pharmacol. Toxicol..

