# Supplementary material for: Overexpression of Neuroglobin Promotes Energy Metabolism and Autophagy Induction in Human Neuroblastoma SH-SY5Y Cells
Source: Cells. 2021 Dec 2;10(12):3394. doi: 10.3390/cells10123394 (PMC8699457; doi:10.3390/cells10123394)
Supplement: Supplementary file 1 [file cells-10-03394-s001.zip › cells-1459469-SI.pdf]

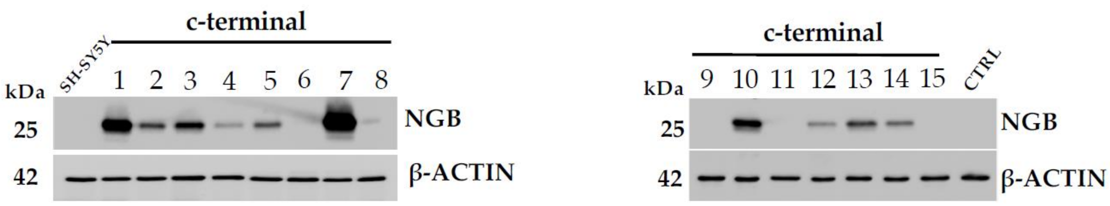

Supplementary Figure S1. Immunoblotting analysis of the cellular clones expressing NGB-FLAG. Neuroblastoma SH-SY5Y cells were transfected with a plasmid leading to the expression of human NGB fused with a FLAG-tag at the C-terminus (NGB-FLAG). SH-SY5Y cells, SH-SY5Y transfected cells with an empty construct (CTRL) and fifteen cellular clones were lysed in RIPA buffer containing 50 mM Tris–HCl pH 7.4, 0.5% Triton X-100, 0.25% Nadeoxycholate, 0.1% SDS, 150 mM NaCl, 1 mM EDTA and 5 mM $MgCl_2$ plus protease inhibitor mixture (Sigma-Aldrich, USA). After evaluation of the protein concentration by Bradford Dye Reagent assay (BioRad, USA), supernatant fraction was separated by 15% SDS-PAGE. For western blotting analysis, polyvinyldene difluoride (PVDF) membranes were incubated with 5% defatted dried milk in Tris-buffered saline (TBS), containing 0.05% Tween 20 and probed with anti-FLAG M2 Clone mAb, (Sigma-Aldrich, USA) or with anti-ACTB (actin, β) mAb, (Sigma-Aldrich). Bound antibodies were visualized with horseradish peroxidase (HRP)-conjugated anti-mouse IgG and immunoreactivity was assessed by chemiluminescence reaction, using the ECL Western detection system (Amersham, UK).

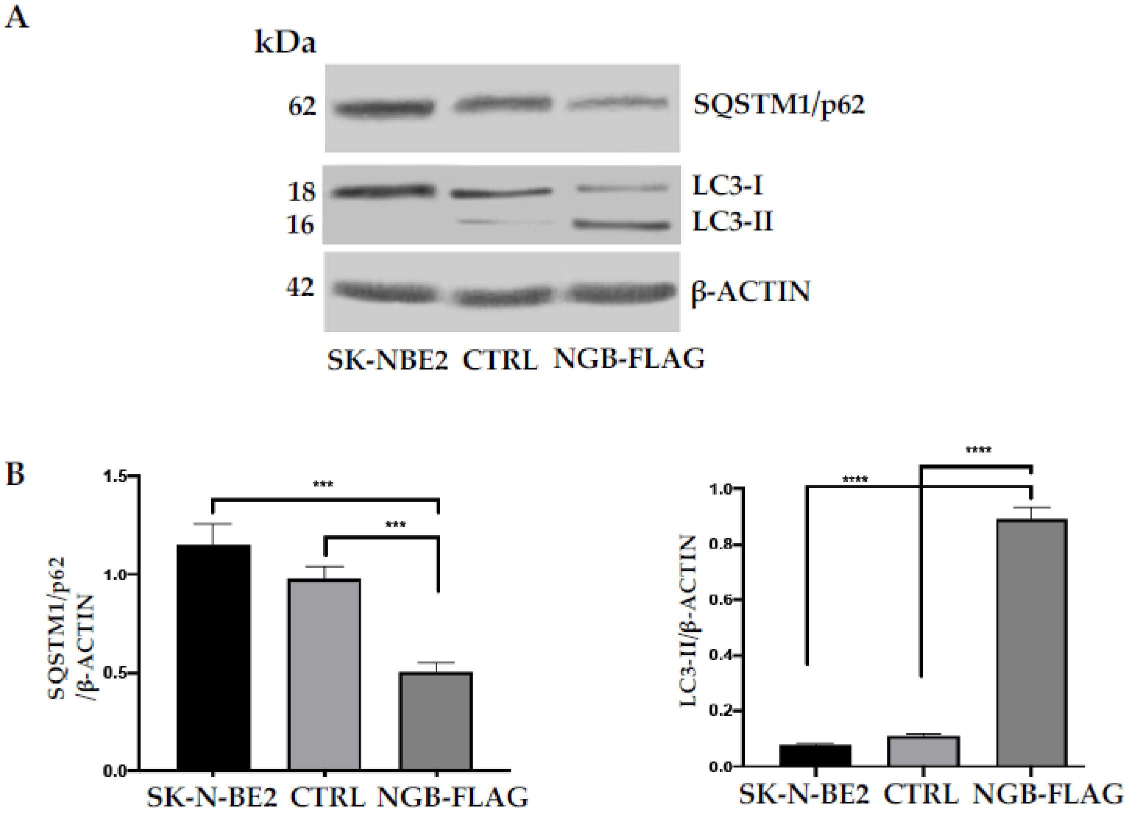

Supplementary Figure S2. Effect of NGB overexpression on autophagy induction in SK-N-BE2 cells. (**A**) Neuroblastoma SK-N-BE2 cells were transfected with a plasmid leading to the expression of human NGB fused with a FLAG-tag (NGB-FLAG) according to Garofalo et al. [16]. SK-N-BE2 cells, SK-N-BE2 transfected cells with an empty construct (CTRL) and stably transfected SK-N-BE2-NGB-FLAG cells were lysed in lysis.buffer, subjected to 15% SDS-PAGE and analyzed by western blot using anti-LC3 polyclonal antibody or rabbit anti-SQSTM1 mAb. Loading control was evaluated using anti-ACTB mAb. A representative experiment among 3 is shown. (**B**) Bar graph on the right shows densitometric analysis. Results represent the mean ± SD from 3 independent experiments. *** $p < 0.001$, **** $p < 0.0001$.
